# Terpinen-4-ol Targets HIF-1α/TGF-β1/TNF-α Axis to Attenuate Ethanol-Induced Hepatotoxicity: Network Pharmacology and In Vitro Validation

**DOI:** 10.3390/medicina61061048

**Published:** 2025-06-06

**Authors:** Tariq G. Alsahli, Maryam Khalid, Muhammad Nasir Hayat Malik, Saud O. Alshammari

**Affiliations:** 1 Department of Pharmacology, College of Pharmacy, Jouf University, Sakaka 72341, Saudi Arabia; tgalsahli@ju.edu.sa; 2 Faculty of Pharmacy, The University of Lahore, Lahore 54000, Pakistan; maryam.khalid@pharm.uol.edu.pk; 3 Department of Pharmacognosy and Alternative Medicine, College of Pharmacy, Northern Border University, Rafha 91911, Saudi Arabia; saud.o.alshammari@nbu.edu.sa

**Keywords:** terpinen-4-ol, HepG2, network pharmacology, HIF-1α, TGF-β1, COL1A1, IL-6

## Abstract

*Background and Objective:* Alcoholic liver disease (ALD) is a major health burden caused by chronic alcohol consumption, leading to oxidative stress, inflammation, and fibrosis. Current treatments are limited, highlighting the need for novel therapeutic agents. This study investigated the hepatoprotective effects of ‘Terpinen-4-ol (T4OL)’, a natural monoterpene from tea tree oil, against ethanol-induced liver injury, focusing on its molecular and cellular mechanisms. *Materials and Methods:* Network pharmacology and molecular docking were employed to predict T4OL’s interaction with ALD-associated targets. Human HepG2 cells were used to validate the in silico findings. Cells were exposed to ethanol (8%) prior to treatment with T4OL or silymarin (SIL), and cytotoxicity was assessed through MTT, crystal violet, and trypan blue assays. Moreover, ELISA and qPCR were conducted to evaluate antioxidant, inflammatory, and fibrotic markers. *Results:* Network pharmacology analysis suggested that T4OL exerts its hepatoprotective effects by suppressing inflammatory and fibrotic mediators (HIF-1α, TGF-β1, and TNF-α). Docking studies also exhibited a strong binding affinity of T4OL to key ALD targets, with docking scores comparable to SIL. In addition, T4OL (13–1300 µM) dose-dependently protected HepG2 cells from ethanol-induced damage, restoring viability by up to 80% at 650 µM. It significantly elevated antioxidant levels (GSH by 2.5-fold, SOD by 1.8-fold) and suppressed pro-inflammatory and fibrotic markers (IL-6, COL1A1, TIMP-1) by 40–60%. At higher concentrations (650–1300 µM), T4OL outperformed SIL in cytoprotection and anti-fibrotic effects. *Conclusions:* T4OL mitigates ethanol-induced liver injury by targeting oxidative stress, inflammation, and fibrosis pathways, demonstrating superior efficacy to SIL at optimal doses. Its multi-target action supports its potential as a therapeutic candidate for ALD.

## 1. Introduction

Hepatotoxicity is described as liver injury or a decline in liver function caused by exposure to xenobiotics [1]. The reported risk factors for hepatotoxicity include the active stage of tuberculosis, old age, female sex, poor nutrition, excessive alcohol consumption, previous history of hepatic illness, hepatitis B virus carrier, hepatic inflammation, improper usage of drugs, and acetylation state [2]. One of the most common liver diseases in both European and American nations is alcoholic liver disease (ALD). Alcohol consumption that surpasses a particular daily limit might cause the illness [3]. Hepatic ethanol metabolism, which normally involves the conversion of ethanol to toxic acetaldehyde, is essential for the development of ALD [4].

The earliest symptoms of ALD include steatosis and minor inflammation. Generally, patients with ALD typically present with clinical manifestations such as asthenia, anorexia, peripheral edema, nausea, emesis, and weight gain. The most frequent clinical assessment is made through the findings of liver function tests [5]. Aspartate transaminase (AST) and alanine transaminase (ALT) ratio is considered an indicator of hepatic illnesses since more than 50% of individuals have an AST/ALT ratio that is two to three times higher than that of a healthy individual. In chronic situations, steatosis can be detected using gamma-glutamyl transpeptidase (GGT), a reliable marker that includes blood cholesterol and lipids levels. However, these markers are unable to discriminate between hepatic failure caused by ethanol and overweight [6].

The most effective treatment approaches for ALD include nutritional therapy [7], alcohol withdrawal therapy [8], and hormone-related therapy. Other targeted therapies include TNF receptor targeting [7], antioxidant signal targeting [9], and inhibiting apoptosis of hepatocytes [10]. Despite significant advancements in understanding the pathophysiology of ALD, effective pharmacological interventions remain limited, with most treatments focusing on alcohol cessation and symptomatic relief. Abstinence dramatically increases survival at any level of hepatic illness, especially cirrhosis. Steatosis returns to normal, and moderate hepatic fibrosis may revert when drinking is avoided. In both symptomatic and non-symptomatic cirrhosis, the mortality rate decreases after alcohol cessation compared to those who continue to drink [4].

In recent times, several medications have gained importance due to their ability to reduce ALD and fibrotic symptoms. These include thalidomide, colchicine, corticosteroids, curcumin, glycyrrhizin, interferons, resveratrol, silymarin (SIL), and sulfoadenosylmethionine [11]. Three newly discovered preventive and curative drugs that are considered effective for the treatment of liver disorders are thalidomide, resveratrol, and curcumin. Sadly, not enough clinical research has been conducted to demonstrate their effectiveness across large populations. Liver transplantation remains the only effective therapy option for patients with end-stage ALD [5,12]. However, it continues to have a number of problems, including a lack of donors, organ rejection, and costly invasive treatment [13]. Existing therapeutic options have demonstrated only modest efficacy and often come with undesirable side effects or limited long-term benefits [12]. Moreover, the growing global burden of ALD and increasing rates of alcohol consumption highlight a pressing need for novel, multi-targeted therapeutic strategies that are both effective and safe.

Natural compounds derived from medicinal plants have lately gained attention due to their broad biological activities and favorable safety profiles [14,15]. Among these, terpinen-4-ol (T4OL), a key component of tea tree oil, has been reported to possess anti-inflammatory and antioxidant properties, both of which are central for ALD therapy [16,17,18,19]. However, the hepatoprotective potential of T4OL in the context of ethanol-induced liver injury remains largely unexplored, particularly with respect to its underlying molecular mechanisms.

To bridge this knowledge gap, this study investigated the hepatoprotective potential of T4OL against ethanol-induced liver toxicity using human HepG2 cells as an in vitro model. By integrating network pharmacology, molecular docking, and functional assays, the research aims to elucidate the underlying mechanisms through which T4OL modulates key signaling pathways associated with oxidative stress, inflammation, and fibrosis. These insights may support the development of T4OL as a promising multi-target therapeutic candidate for ALD.

## 2. Materials and Methods

### 2.1. Chemicals

SIL, T4OL, Dulbecco’s Modified Eagle’s Medium (DMEM), and Pen-Strep were procured from Sigma-Aldrich, St. Louis, MO, USA. All other consumables used in this research were of standard quality.

### 2.2. In Silico Study—Network Pharmacology Analysis

#### 2.2.1. T4OL and ALD-Associated Targets Prediction

We used data from four different databases to determine the potential biological targets. Swiss Target Prediction employs a comprehensive library of approximately 370,000 bioactive compounds to predict potential targets across more than 3000 proteins from three different species [20]. Way2Drug (DIGEP-Pred 2.0) is an online platform that forecasts drug-induced alterations in gene expression based on the structural formulas of small-molecule compounds. It integrates gene expression data from the Comparative Toxicogenomics Database (CTD) at both the mRNA and protein levels, along with insights from the Connectivity Map (CMAP) build02 [21]. Way2Drug (Pass online) is an online web server tool that predicts the biological activity spectra for small organic and inorganic molecules. It employs a unique approach called Prediction of Activity Spectra for Substances (PASS) to model and anticipate a molecule’s pharmacological properties and biological targets. Way2Drug (Pass target) predicts its probable activities and the inactive state towards different biological targets like enzymes, receptors, and transporters [22]. Super-PRED is a sophisticated web server designed for predicting the Anatomical Therapeutic Chemical (ATC) codes and molecular targets of small compounds by utilizing machine learning techniques, specifically logistic regression and Morgan fingerprints based on their therapeutic and chemical properties [23]. A publicly available web server called PharmMapper employs a pharmacophore mapping technique, which entails matching the spatial arrangement of characteristics necessary for a chemical to interact with a particular target receptor [24]. GeneCards is an integrative database that compiles gene-related information from over 150 online sources, encompassing genomic, transcriptomic, proteomic, genetic, clinical, and functional data specific to human genes. To construct a target profile for T4OL, retrieved targets were consolidated, and duplicate entries were eliminated to ensure dataset integrity.

For identifying targets associated with ALD, data were collected from GeneCards, the CTD, and the Open Targets Platform (OTP). These targets were filtered based on gene-disease association scores, and only *Homo sapiens* proteins were considered. CTD serves as a curated repository of chemical–gene–disease interactions, encompassing over 49,000 genes and 13,000 diseases. Meanwhile, the OTP database facilitates therapeutic target identification by systematically scoring gene-disease associations, incorporating data on more than 21,000 genes and 10,000 disease conditions [25].

#### 2.2.2. Gene Matching and Venn Diagram Construction

We used the SRplot online platform (https://www.bioinformatics.com.cn/en) to identify shared genes between the disease and the compound. The SR plot was used to show the overlap of the gene sets. This plot identified common genes that may be involved in the therapeutic efficacy of T4OL [26,27].

#### 2.2.3. Protein–Protein Interaction (PPI) Network

The screened molecular targets were mapped and analyzed using Cytoscape version 3.10.2, an open-source software platform designed for the construction, visualization, and interpretation of complex biological interaction networks. Cytoscape facilitates the identification of protein targets, their interactions with compounds, and their involvement in disease-associated pathways [28]. To predict protein–protein interactions (PPIs) among the hub genes, the STRING database was employed, focusing specifically on interactions in Homo sapiens with a high confidence score threshold of ≥0.9. The resulting PPI data were subsequently imported into Cytoscape for network visualization. Within the constructed network, key hub proteins were identified and ranked based on betweenness centrality (BC)—a critical topological parameter that quantifies the influence of a node in regulating communication across the network [29].

#### 2.2.4. Gene Ontology and Functional Enrichment Analysis

Gene Ontology (GO) enrichment analysis was performed using the DAVID platform, encompassing four major annotation categories: KEGG pathways, biological processes (BPs), cellular components (CCs), and molecular functions (MFs). To visually present the enrichment results, the SRplot web platform was utilized, generating both bubble and bar plots that effectively illustrated the distribution and significance of enriched terms, highlighting SRplot’s utility as a versatile tool for graphical data representation [30].

### 2.3. Molecular Docking

#### 2.3.1. Preparation of Ligand and Target Proteins

The 2D structure of T4OL was initially generated using ChemDraw, version 22.0.0.22 (Figure S1). Its corresponding 3D structure was retrieved from the PubChem database, maintained by the National Center for Biotechnology Information (NCBI), and subsequently converted into Protein Data Bank (PDB) format using BIOVIA Discovery Studio Visualizer. Prior to docking studies, the ligand was preprocessed in MOE (Molecular Operating Environment) to eliminate counterions and salts and to assign an appropriate protonation state. The structure was then subjected to energy minimization using the Merck Molecular Force Field (MMFF94x) to ensure the most stable and low-energy conformation was achieved for accurate molecular interaction analysis [31].

The 3D structures of protein targets, i.e., human IKB-α/NF-κB1 complex (PDB ID: 1IKN) classified as transcription factor at a resolution rate of 2.30 Å, human hypoxia-inducible factor-1 alpha (HIF-1α; PDB ID: 1H2M) classified as transcription activator/inhibitor at a resolution of 2.50 Å, human tumor necrosis factor-alpha (TNF-α; PDB ID: 2AZ5) classified as cytokine at a resolution of 2.10 Å, human collagen alpha-1(I) chain (COL1A1; PDB ID: 5K31) classified as structural protein at a resolution of 2.20 Å, human matrix metllaoproteinase-1 (MMP1; PDB ID: 3SHI) classified as hydrolase at a resolution of 2.20 Å, and human matrix metalloproteinase-3/tissue inhibitor of metalloproteinase-1 complex (MMP-3/TIMP-1 complex; PDB ID: 1UEA) classified as complex (metalloprotease/inhibitor) at a resolution of 2.80 Å, determined through X-ray diffraction, were obtained from RCSB in PDB format [32,33]. The MOE Protonate-3D module preprocessed the protein structures to ensure their docking analysis (Figure S2) [32,33].

#### 2.3.2. Active Binding Site Estimation

CASTp was used to predict the active binding sites of different proteins [34]. The predicted binding sites are listed in Table S1.

#### 2.3.3. Docking of Ligand with Target Proteins

MOE (version 2019.0102) was used to calculate molecular docking and score calculations. The BIOVIA Discovery Studio 2024 client, version 24.1 was used to visualize the docking analysis that was carried out for ligands against various protein targets. Initially, the ligand database was constructed using MOE and converted into a Microsoft Access database (MDB) format for compatibility with the docking workflow. Docking simulations were then performed using MOE’s default docking protocol, which employed the Triangle Matcher algorithm for pose generation, along with London dG and GBVI/WSA dG scoring functions for preliminary and refined scoring, respectively. A total of 50 ligand conformations were evaluated, and the optimal binding pose was identified based on scoring metrics and visualized using BIOVIA Discovery Studio, ensuring an accurate assessment of molecular interactions.

### 2.4. HepG2 Cell Culture

The University of Lahore’s “Cell and Tissue Culture Laboratory” provided the HepG2 cell line. The cells were grown at 37 °C in DMEM (Cat. N0. D5030) (Sigma Aldrich, St. Louis, MO, USA) supplemented with 100 g/mL streptomycin (Cat. No. S9137), 100 units/mL penicillin (Cat. No. P3032), and 10% fetal bovine serum (Cat. No. F4135) [35].

#### 2.4.1. Cytotoxic Evaluation of T4OL and Ethanol

For ethanol cytotoxicity testing, various doses (1–10%) of ethanol were formulated in growth medium. Moreover, a stock solution of T4OL (0.013 M) was prepared in 1% DMSO, and various dilutions of T4OL (13, 130, 650, 1300, 2600 µM) from the stock solution were prepared in complete DMEM (growth) medium. HepG2 cells were grown in a 96-well plate, which was later placed in an incubator. After overnight incubation, the medium was removed, and cells were washed with 1X PBS. A total of 100 µL of various T4OL and ethanol dilutions was added to each well. Cells were incubated for 24 h, and cell viability was assessed by MTT (3-[4,5-dimethylthiazol-2-yl]-2,5 diphenyl tetrazolium bromide) and crystal violet assays [35,36].

#### 2.4.2. Determination of Hepatoprotective Effect of T4OL

HepG2 cells were grown in a 96-well plate for cell viability assays and a 6-well plate for ELISA, antioxidant, and gene expression experiments. Cells were pre-treated for 24 h with 100 µL of different doses of T4OL. Subsequently, cells were rinsed with PBS, and 8% ethanol was applied for 24 h to induce injury. For purposes of comparison, SIL (200 g/mL) was used as the standard drug [35,36].

The following treatment groups were used in this study (n = 4 in each group):

1. Control—Complete growth medium;
2. Disease group—8% ethanol in complete growth medium;
3. SIL 200 µg/mL—200 µg/mL of SIL in complete growth medium;
4. T4OL (13 µM)—13 µM of T4OL in complete growth medium;
5. T4OL (130 µM)—130 µM of T4OL in complete growth medium;
6. T4OL (650 µM)—650 µM of T4OL in complete growth medium;
7. T4OL (1.3 mM)—1.3 mM of T4OL in complete growth medium.

### 2.5. Cell Viability Assays

To determine the viability of treated HepG2 cells, MTT (3-[4,5-dimethylthiazol-2-yl]-2,5 diphenyl tetrazolium bromide) and crystal violet assays were carried out on cells cultivated in 96-well plates using various concentrations of the aforementioned dilutions.

#### 2.5.1. MTT Assay

T4OL and ethanol were used to treat the cells, after which they were rinsed with phosphate-buffered saline (PBS). The cells were later incubated for 3–4 h with 100 µL of growth medium and 25 µL of MTT solution. After 4 h, 10% sodium dodecyl sulfate (SDS) was added to the formazan crystals, and the absorbance was measured at 570 nm [35].

#### 2.5.2. Crystal Violet Test

The medium was removed from the wells, and the cells were rinsed with PBS. After washing, the wells were filled with crystal violet dye and incubated at room temperature for 15 min. The wells were properly washed with PBS in order to prevent the cells from lifting out of the well. Then, 100 µL of 1% SDS was added to each well, and the cells were left for 5 to 10 min. Finally, a microplate reader was used to measure the absorbance at 595 nm [35].

### 2.6. Antioxidant Assays

#### 2.6.1. Glutathione Reductase (GSH) Assay

The GSH content was determined by the Bioassay Technology Laboratory ELISA Kit (Cat. No. EA0142Hu; Shanghai, China). All the reagents, standard solutions, and samples were prepared according to the instructions and brought to room temperature. Every standard well received 50 µL of standard, while every sample well received 40 µL of sample. Each well received 10 µL of anti-GSH antibody and 50 µL of streptavidin-HRP sample. The plate was later sealed with a sealer and allowed to sit at 37 °C for 60 min. Following the incubation period, the sealer was taken off, and the plate underwent five washes with wash buffer. A total of 300 µL of wash buffer was poured into each well and left for 30 s to 1 min. Subsequently, each well received 50 µL of substrate solution A followed by 50 µL of substrate solution B. The plate was then incubated for 10 min at 37 °C in the dark. Once the incubation period was over, stop solution was added, and absorbance at 450 nm was measured using a microplate reader (Bio-Rad microplate ELISA reader, PR4100, Hercules, CA, USA) [37].

#### 2.6.2. Superoxide Dismutase (SOD) Assay

SOD was assessed using the same protocol as the GSH assay using Bioassay Technology Laboratory ELISA Kit (Cat. No. E4502Hu; Shanghai, China).

Similarly, the levels of specific proteins (NF-κB1, HIF-1α, IL-6, TGF-β1, COL1A1, MMP1, and TIMP1) in the cell culture supernatants were quantified using commercially available ELISA kits.

#### 2.6.3. Real-Time PCR Analysis

Total RNA was extracted using TRIzol reagent, followed by reverse transcription into complementary DNA (cDNA) using the WizScript™ cDNA Synthesis Kit (Wizbio Solutions, Loco Hills, NM, USA; Cat. No. W2202), according to the manufacturer’s instructions. Gene expression analysis was conducted using the Applied Biosystems StepOne™ (Thermo Fischer, Waltham, MA, USA) Real-Time PCR System in combination with Zokeyo 2x SYBR Green qPCR Mixture (Zokeyo, Wuhan, China; Cat. No. HPR012-01). The expression levels of target genes were quantified, and hypoxanthine-guanine phosphoribosyltransferase (HPRT) served as the internal reference gene for normalization.

### 2.7. Statistical Analysis

The findings were displayed as mean ± standard deviation, and Graph Pad Prism 8.0 was used to analyze the biological replicate data using one-way ANOVA and Tukey’s multiple comparison test. Probability values of less than 0.05 were deemed as significant. The following symbols were used to denote significance: *** ≤ 0.001, ** ≤ 0.01, * ≤ 0.05.

## 3. Results

### 3.1. Prediction of the Biological Spectrum

The biological activity spectrum of T4OL was predicted using the Way2Drug PASS Online database, which estimates the probable pharmacological effects based on the compound’s structural properties. A total of four proteins were selected based on their significant down/upregulation. The “Probable activity (Pa)” and “probable inactivity (Pi)” were the letters used to indicate the likely functions. The actions related to Pa > Pi were considered in the current analysis of biological spectrum interpretation. T4OL demonstrated inhibitory activity against TNF, NF-κB, HIF-1α, and TGF-β1.

### 3.2. Strong Binding Interactions of T4OL with Target Proteins

**Figure 1:**
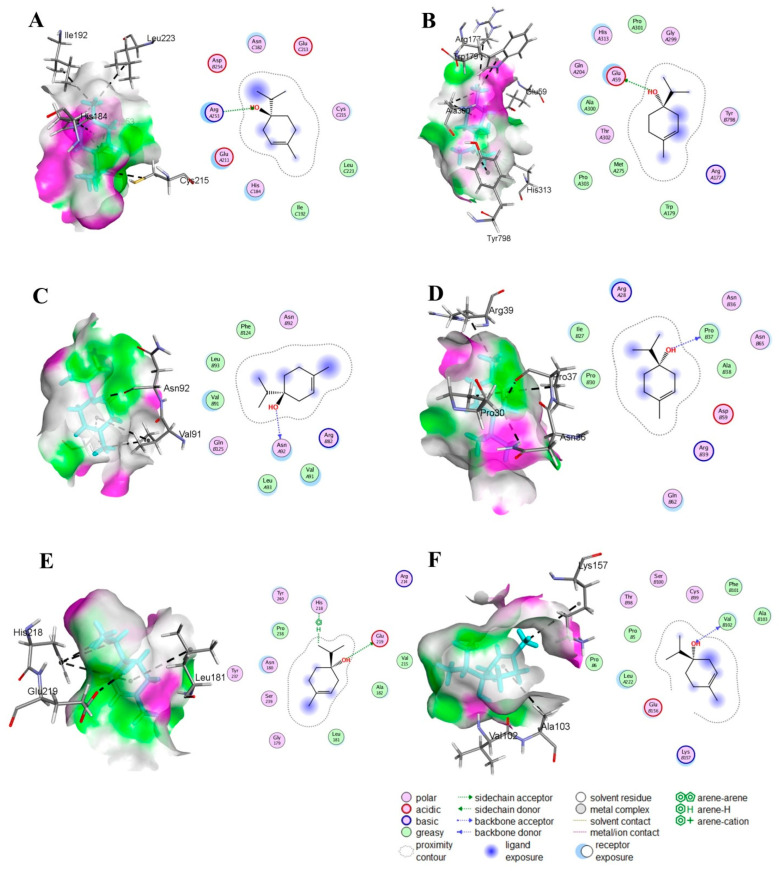
Molecular docking of T4OL by Discovery Studio. Two-dimensional illustration of several linkages attached to important amino acids. The ligands are displayed as cyan sticks with surrounding residues, and interaction bonds are depicted as black dashed lines. In silico interactions of T4OL with different proteins: (**A**) human NF-κB complex; (**B**) human HIF-1α; (**C**) human TNF-α; (**D**) human COL1A1; (**E**) human MMP-1; (**F**) human TIMP-1.

The molecular docking studies conducted for T4OL and SIL against various proteins indicated that both the compounds exhibited robust interactions with several proteins relevant to ALD, including NF-κB, HIF-1α, TNF-α, COL1A1, MMP-1, and TIMP-1. The binding affinities of T4OL were comparable with SIL across all targets. The binding scores of SIL ranged from −6.52 to −8.42 kcal/mol, while T4OL’s scores were slightly lower, ranging from −4.63 to −5.40 kcal/mol. These results were supported by RMSD values below 2 Å for both compounds, indicating stable docking conformations. Key interactions included the formation of hydrogen bonds and pi-H interactions with critical amino acid residues, such as the binding of T4OL to ARG 253 in NF-κB and GLU 59 in HIF-1α, ASN 92 in TNF-α, PRO 37 in COL1A1, GLU 219 and HIS 218 in MMP-1, VAL 102 in TIMP-1 (Table 1; Figure 1). The stronger binding affinity and multiple interactions with different residues against targets for T4OL suggested it may effectively modulate these proteins, potentially leading to greater reductions in inflammation, fibrosis, and other pathological processes associated with ALD.

### 3.3. Target Proteins of T4OL Linked with ALD

**Figure 2:**
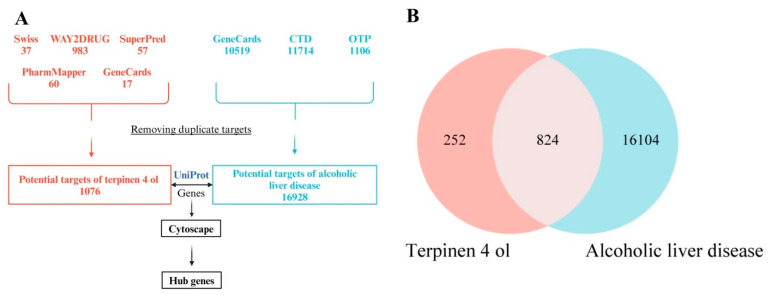
Screening of T4OL targets relevant to ALD. (**A**) Flowchart illustrating the identification of core molecular targets of T4OL and ALD through the integration of data from multiple databases. (**B**) Venn diagram showing the intersection of target genes associated with T4OL and ALD. The red circle represents unique molecular targets of T4OL, while the light blue circle indicates targets uniquely associated with ALD. The overlapping region denotes 824 common targets shared between T4OL and ALD.

**Figure 3:**
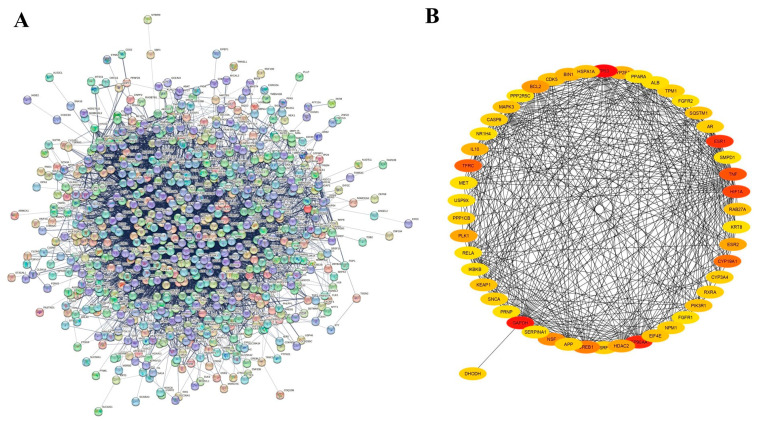
PPI network of intersected target genes. (**A**) PPI network of T4OL-ALD molecular target using STRING, which comprises 814 nodes and 7533 edges. Potential 814 unique core targets identification between 1076 T4OL-related proteins and 16,928 proteins related to ALD. (**B**) Topmost 50 target proteins based on betweenness centrality generated by Cytoscape 3.10.2.

A total of 1076 targets of T4OL were predicted, while 16,928 possible targets were identified for ALD from different databases. There were 824 matching genes shown by the Venn diagram and 814 unique targets where T4OL and ALD targets intersected in the STRING database, which comprises 814 nodes, 7533 edges, and PPI enrichment *p*-value < 1.0 × 10^−16^. The top 10 targets of T4OL against ALD included tumor protein 53, glyceraldehyde-3-phosphate dehydrogenase, heat shock protein 90-α A1, estrogen receptor 1, HIF-1α, TNF-α, transferrin receptor, cytochrome P450 19 A1, cAMP responsive element binding protein 1, and N-ethylmaleimide sensitive factor (Figure 2 and Figure 3).

### 3.4. GO Analysis

**Figure 4:**
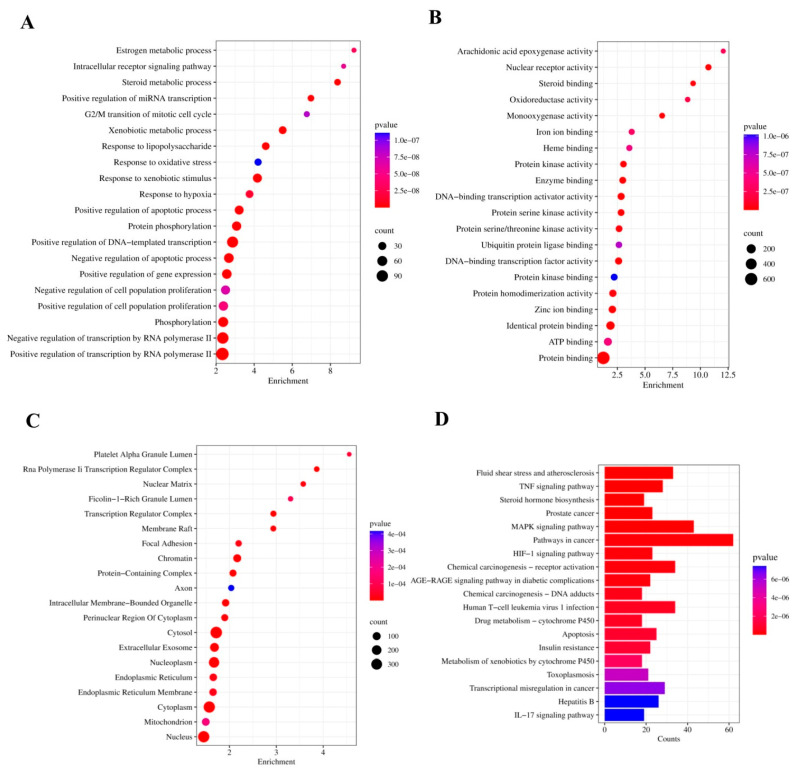
Functional annotation and enriched pathways associated with T4OL in the context of ALD: (**A**) biological processes (BPs), (**B**) molecular functions (MFs), and (**C**) cellular components (CCs) identified via GO enrichment analysis. The size of each bubble reflects the number of genes enriched within the corresponding GO term, with larger bubbles indicating greater gene involvement. (**D**) Top 20 KEGG pathways enriched among T4OL-associated targets. The color gradient of each bar represents the adjusted *p*-value, where a deeper blue indicates a lower significance level. Visualization of both bubble and bar plots was performed using the SRplot platform.

GO function analysis identified BP, CC, and MF entries for hub genes. The topmost 10 entries of BP included positive regulation of transcription by RNA polymerase II, positive regulation of DNA-templated transcription, response to xenobiotic stimulus, negative regulation of transcription by RNA polymerase II, xenobiotic metabolic process, protein phosphorylation, steroid metabolic process, response to hypoxia, and response to oxidative stress. The topmost 10 entries of MF included protein binding, nuclear receptor activity, identical protein binding, DNA-binding transcription activator activity, DNA-binding transcription factor activity, enzyme binding, protein serine kinase activity, zinc ion binding, monooxygenase activity, and protein kinase activity. The topmost entries of CC included cytosol, cytoplasm, nucleoplasm, nucleus, chromatin, extracellular exosome, intracellular membrane-bounded organelle, protein-containing complex, transcription regulator complex, and RNA polymerase II transcription regulator complex. A KEGG pathway enrichment analysis identified 143 linked signaling pathways. The topmost 10 KEGG enrichment analysis identified the involvement of pathways including lipid and atherosclerosis (hsa05417), fluid shear stress and atherosclerosis (hsa05418), TNF signaling pathway (hsa04668), steroid hormone biosynthesis (hsa00140), prostate cancer (hsa05215), MAPK signaling pathway (hsa04010), pathways in cancer (hsa05200), HIF-1 signaling pathway (hsa04066), chemical carcinogenesis—receptor activation (hsa05207), and AGE-RAGE signaling pathway in diabetic complications (hsa04933) (Figure 4).

### 3.5. Cytotoxic Dose Determination of Ethanol and T4OL by MTT Assay

**Figure 5:**
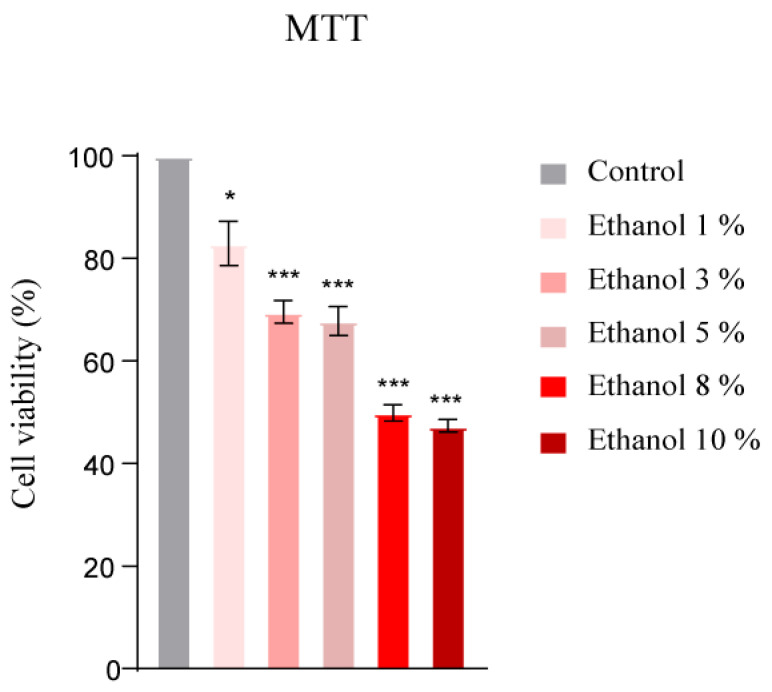
Dose-dependent reduction in cell viability by ethanol. A concentration-dependent decrease in cell viability was observed with higher ethanol exposure. One-way ANOVA followed by Tukey’s multiple comparison tests (n = 4); *** ≤ 0.001, * ≤ 0.05.

The cytotoxic effects of T4OL and ethanol were investigated in HepG2 cells prior to hepatoprotective experiments. HepG2 cells were treated with varying concentrations of ethanol for 24 h to evaluate its impact on cell viability. The results demonstrated that ethanol significantly reduced cell viability at higher doses of 8% and 10%, as shown in Figure 5. This dose-dependent cytotoxicity highlighted the need to carefully consider ethanol concentrations in experimental setups.

For T4OL, the cytotoxicity assessment revealed no adverse effects on cell viability at concentrations of 13, 130, 650, and 1300 µM, indicating these doses were well-tolerated by HepG2 cells. However, at the highest tested concentration of 2.6 mM (2600 µM), T4OL significantly decreased cell viability, suggesting potential toxicity at this level. Based on these findings, the 2.6 mM concentration of T4OL was excluded from hepatoprotective experiments to avoid confounding results due to its cytotoxic effects. Consequently, lower concentrations of T4OL, which demonstrated no significant impact on cell viability, were selected for subsequent evaluations to ensure the reliability and safety of the hepatoprotective study outcomes (Figure S3).

### 3.6. T4OL’s Hepatoprotective Response to Ethanol-Induced Cellular Damage

**Figure 6:**
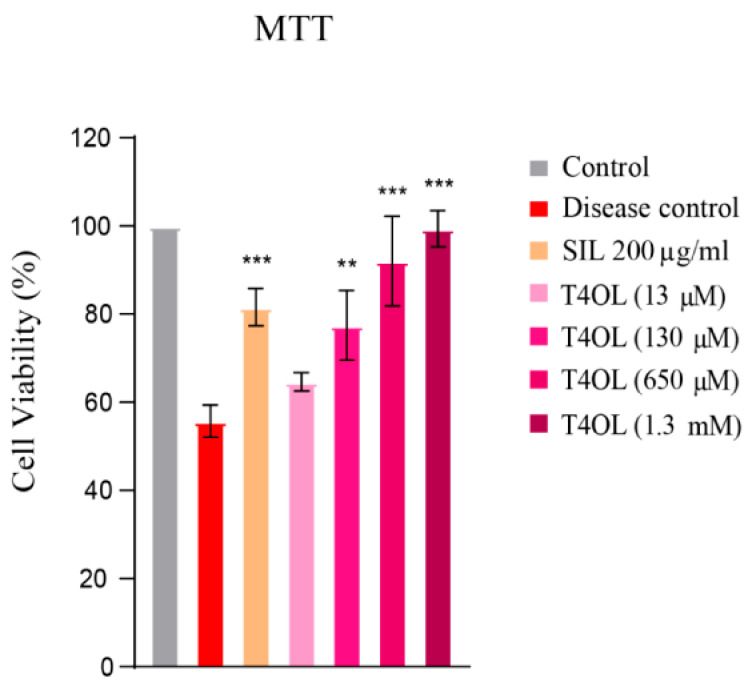
MTT assay depicting hepatoprotective effect of T4OL. Cells were pre-treated with T4OL, followed by intoxication with 8% ethanol. T4OL dose-dependently protected against ethanol-induced injury. One-way ANOVA and post hoc test, n = 4, *** ≤ 0.001, ** ≤ 0.01.

The MTT assay results highlighted the dose-dependent hepatoprotective effects of T4OL against ethanol-induced cytotoxicity in HepG2 cells. As the concentration of T4OL increased, its ability to mitigate the harmful effects of ethanol became more pronounced. Notably, at higher concentrations of 650 and 1300 µM, T4OL exhibited superior hepatoprotective efficacy compared to the widely used standard drug, SIL, a benchmark treatment for liver toxicity. This significant observation, illustrated in Figure 6, underscores the potential of T4OL as an effective agent for safeguarding liver cells from ethanol-induced damage.

Furthermore, the crystal violet assay provided consistent results, reinforcing the findings from the MTT assay. This complementary assay also confirmed that T4OL’s hepatoprotective effects were dose-dependent and more prominent at higher concentrations. At 650 and 1300 µM, T4OL outperformed SIL in protecting against ethanol-induced damage, suggesting that it may serve as a more effective therapeutic option for liver protection. The congruence between the MTT and crystal violet assays strengthens the evidence supporting T4OL’s dose-dependent hepatoprotective properties and highlights its promise as a potent alternative to existing treatments for ethanol-related hepatotoxicity (Figure S4).

### 3.7. T4OL Restored GSH and SOD Levels

This study demonstrated that ethanol exposure in HepG2 cells led to a significant reduction in the levels of crucial antioxidants (GSH and SOD). These antioxidants play a vital role in maintaining cellular redox balance and protecting cells from oxidative damage. The decline in their levels following ethanol treatment indicates the onset of oxidative stress, which is a key factor contributing to ethanol-induced cytotoxicity and liver injury. The depletion of GSH and SOD highlights the compromised antioxidant defense system in ethanol-exposed cells, leaving them more vulnerable to oxidative damage.

**Figure 7:**
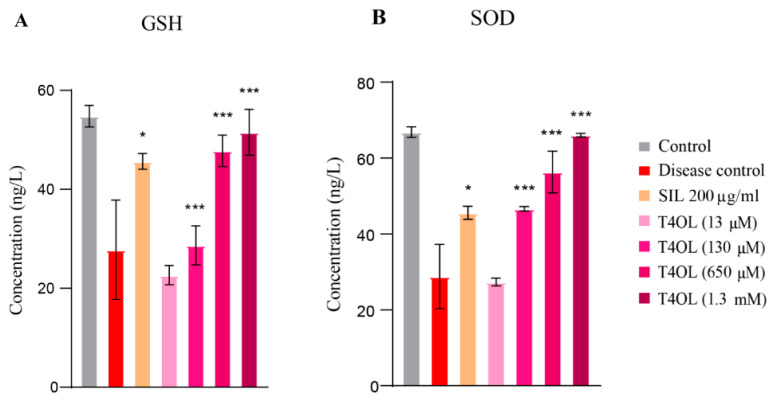
T4OL enhanced the levels of GSH and SOD. (**A**) GSH and (**B**) SOD levels measured as concentration. Cells were pre-treated with T4OL, followed by intoxication with 8% ethanol. GSH and SOD levels were measured by ELISA using supernatants. T4OL significantly increased the levels of antioxidants as compared to the disease group. One-way ANOVA and post hoc test, n = 4, *** ≤ 0.001, * ≤ 0.05.

Interestingly, treatment with T4OL effectively reversed this trend by dose-dependently enhancing the levels of both GSH and SOD. This suggests that T4OL has a strong antioxidative capacity, helping to restore the cellular antioxidant defense system disrupted by ethanol. At higher concentrations of T4OL, specifically 650 and 1300 µM, the restoration of GSH and SOD levels was particularly notable. The antioxidant levels at these doses were comparable to those observed in the SIL-treated group. These findings emphasize the ability of T4OL to mitigate ethanol-induced oxidative stress by replenishing antioxidant reserves and enhancing cellular resilience. The dose-dependent effect of T4OL further suggests that higher concentrations may provide optimal protection against oxidative damage. This positions T4OL as a promising therapeutic candidate for managing ethanol-induced liver injury, with effects comparable to the well-established hepatoprotective agent SIL, particularly at elevated doses (Figure 7).

### 3.8. T4OL Reduced mRNA Levels of Inflammatory and Fibrotic Markers

The findings of this study demonstrated that treatment with T4OL significantly reduced the relative transcript levels of key molecular markers associated with oxidative stress, inflammation, and fibrosis. These markers included COL1A1, glutathione peroxidase-7 (GPX-7), TGF-β1, IL-6, MMP1, and TIMP-1. In the ethanol-treated disease group, the expression of these biomarkers was markedly elevated, reflecting the pathological mechanisms triggered by ethanol exposure.

Ethanol-induced liver injury is characterized by a cascade of interconnected processes. Oxidative stress, driven by an imbalance in reactive oxygen species (ROS) and antioxidant defenses, is indicated by elevated levels of GPX-7. Inflammatory responses, marked by increased IL-6 expression, further exacerbate tissue damage. Simultaneously, ethanol exposure promotes fibrotic processes, evidenced by heightened expression of COL1A1 (a major component of collagen deposition), TGF-β1 (a pivotal regulator of fibrosis), and dysregulation of the MMP1–TIMP-1 axis, which governs extracellular matrix remodeling and fibrosis progression.

**Figure 8:**
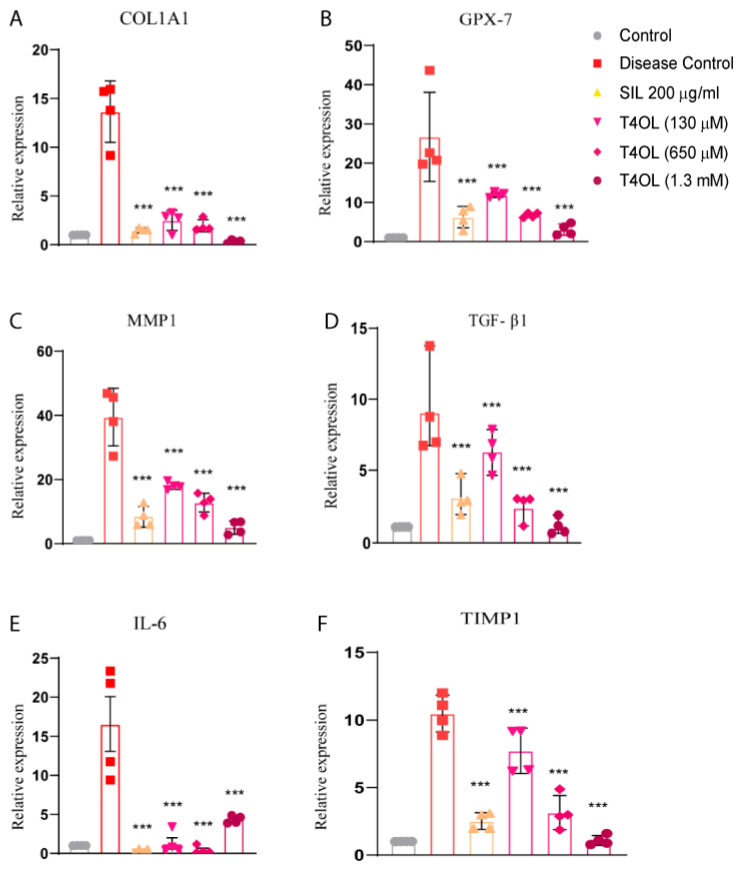
T4OL reduced the expression of ethanol-induced pro-fibrotic and inflammatory biomarkers. Cells were pre-treated with T4OL, followed by intoxication with 8% ethanol. Relative mRNA levels of (**A**) *COL1A1*, (**B**) *GPX-7*, (**C**) *MMP1*, (**D**) *TGF-β1*, (**E**) *IL-6*, and (**F**) *TIMP-1* were measured in experimental groups including Disease Control, SIL (200 μg/mL), and T4OL-treated samples (130 μM, 650 μM, and 1.3 mM). T4OL significantly reduced the expression levels of these biomarkers as compared to the disease group. One-way ANOVA and post hoc test, n = 4, *** ≤ 0.001.

Treatment with T4OL effectively suppressed these pathological mechanisms by significantly downregulating the expression of these biomarkers. The reduction in GPX-7 levels suggests that T4OL mitigates oxidative stress, potentially by restoring redox balance. The decreased expression of IL-6 indicates an attenuation of inflammatory signaling, helping to alleviate inflammation-induced tissue damage. Furthermore, T4OL’s ability to lower the transcript levels of COL1A1, TGF-β1, MMP1, and TIMP-1 highlights its anti-fibrotic effects, suggesting a protective role against collagen deposition and fibrotic scarring. These results suggest that T4OL exerts a multifaceted protective effect against ethanol-induced liver damage by targeting the core mechanisms of oxidative stress, inflammation, and fibrosis (Figure 8).

### 3.9. Dose-Dependent Amelioration of Alcohol-Induced Liver Injury by T40L via Inhibition of Inflammatory, Hypoxic, and Fibrotic Signaling

The present ELISA findings illustrate a comprehensive evaluation of the modulatory effects of T4OL on critical molecular markers involved in inflammatory and fibrotic pathways associated with ALD. Beginning with NF-κB1 expression, the Disease Control group showed a dramatic increase over baseline, confirming inflammatory pathway activation. T4OL produced a progressive, concentration-dependent suppression of NF-κB1, with the 1.3 mM dose reducing levels to those approaching the Control. This pattern suggests potent inhibition of canonical NF-κB signaling. The adjacent graph depicting HIF-1α levels revealed pathological accumulation in the Disease Control group, consistent with oxidative stress. T4OL treatment elicited a striking dose-responsive decrease, particularly at 650 μM and 1.3 mM concentrations.

**Figure 9:**
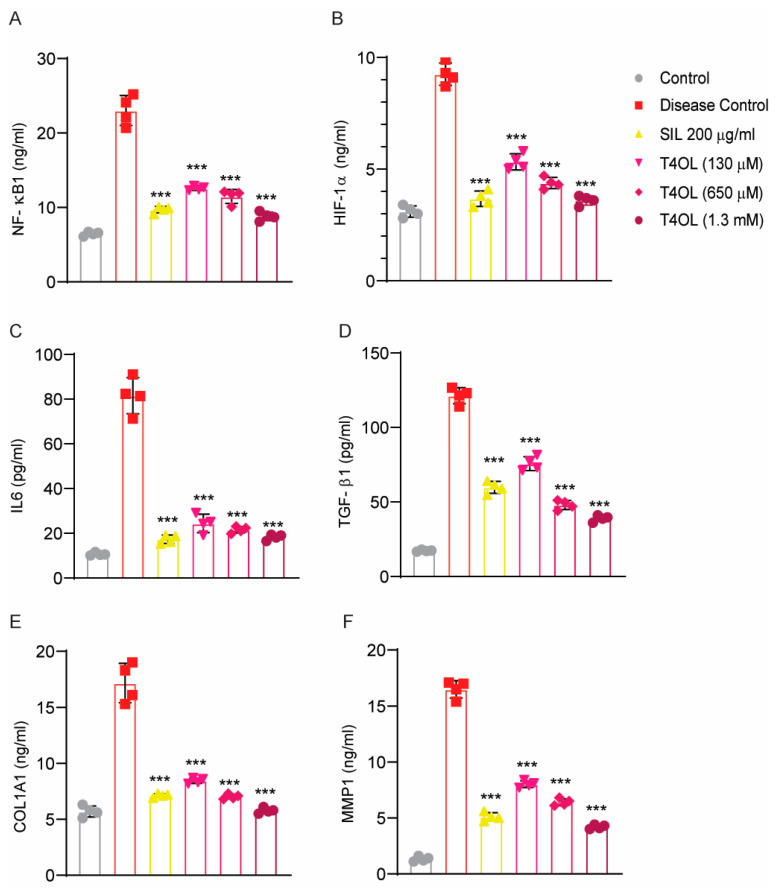
Therapeutic effects of T4OL on inflammatory, hypoxic, and fibrotic markers in an experimental model of ALD. Bar graphs depict the concentrations of (**A**) NF-κB1 (ng/mL), (**B**) HIF-1α (ng/mL), (**C**) IL-6 (pg/mL), (**D**) TGF-β1 (pg/mL), (**E**) COL1A1 and (**F**) MMP1 (ng/mL) across treatment groups: Control (healthy baseline), Disease Control (untreated ALD model), SIL (200 μg/mL reference treatment), and T4OL at three doses (130 μM, 650 μM, 1.3 mM). T4OL demonstrated dose-dependent suppression of pro-inflammatory (NF-κB1, IL-6), hypoxic (HIF-1α), and pro-fibrotic (TGF-β1) markers while restoring physiological MMP1 levels, suggesting multitargeted efficacy against ALD pathogenesis. One-way ANOVA and post hoc test, n = 4, *** ≤ 0.001.

For IL-6 secretion, the Disease Control group exhibited the expected inflammatory surge, which is markedly attenuated by all active treatments. The near-complete normalization with high-dose T4OL (1.3 mM) underscores its capacity to disrupt cytokine amplification loops, likely secondary to upstream NF-κB inhibition. Finally, MMP1 dysregulation in the Disease Control group is rectified by T4OL in a concentration-dependent manner. MMP1 is an extracellular matrix degrading enzyme and its increase in the disease group could be due to ethanol-induced oxidative stress and inflammation. The restoration of physiological MMP1 levels at 1.3 mM indicated rebalancing of extracellular matrix turnover—a critical determinant of tissue remodeling outcomes (Figure 9).

The TGF-β1 data demonstrated the compound’s anti-fibrotic potential, with T4OL progressively blunting the Disease Control group’s elevated levels. The maximal effect at 1.3 mM suggests possible interference with TGF-β receptor dimerization, Smad2/3 phosphorylation, or non-canonical signaling through MAPK pathways (Figure 9).

The multitargeted effects of T4OL—simultaneously reducing inflammation (NF-κB1/IL-6), hypoxia (HIF-1α), and fibrosis (TGF-β1/MMP1)—make it a promising candidate for ALD treatment, particularly in alcoholic-steatohepatitis and early fibrosis stages. Compared to SIL, which showed partial efficacy, T4OL’s dose-dependent response suggests a wider therapeutic window, potentially offering greater clinical benefit.

## 4. Discussion

ALD is a major global health concern, characterized by a progression from simple steatosis to steatohepatitis, fibrosis, and eventually cirrhosis or hepatocellular carcinoma, depending on the severity and duration of ethanol exposure [38]. The pathogenesis of ALD is multifactorial, involving oxidative stress, inflammatory signaling, and excessive extracellular matrix (ECM) deposition. Hepatocellular injury resulting from ethanol metabolism is driven by the generation of ROS, mitochondrial dysfunction, and immune-mediated damage [36,39,40]. This study aimed to explore the therapeutic potential of T4OL in mitigating these pathological hallmarks by examining its impact on the expression of critical molecular markers involved in ALD.

Among the fibrotic markers evaluated, COL1A1 plays a central role in liver fibrosis by encoding a major fibrillar collagen involved in ECM deposition. Collagen I is produced primarily by activated hepatic stellate cells, which transdifferentiate into myofibroblasts upon liver injury and contribute to fibrotic remodeling [41]. Excessive accumulation of COL1A1 distorts liver architecture, impairs function, and marks the transition to advanced fibrosis and cirrhosis [42]. In our model, ethanol exposure induced upregulation of COL1A1, while T4OL treatment significantly suppressed its expression in a dose-dependent manner. This finding suggests that T4OL exerts anti-fibrotic effects by interfering with HSC activation or ECM gene transcription.

TGF-β1 is a master regulator of fibrosis and a potent inducer of COL1A1 expression. Upon activation by ethanol-induced ROS, TGF-β1 binds to its receptors and triggers Smad signaling pathways, promoting transcription of pro-fibrogenic genes, including those for collagens and tissue inhibitors of metalloproteinases [43]. Elevated TGF-β1 levels are directly correlated with liver fibrosis severity and have been identified as therapeutic targets in ALD and non-alcoholic steatohepatitis (NASH) [44]. Our results demonstrate that T4OL downregulates TGF-β1 expression, thereby likely attenuating downstream pro-fibrotic signaling cascades and impeding fibrogenesis at its source.

TIMP-1 complements TGF-β1 in fibrotic progression by inhibiting matrix metalloproteinases (MMPs), particularly MMP1, which degrade ECM proteins such as type I collagen. An imbalance between MMPs and TIMPs skews ECM homeostasis toward matrix accumulation [45]. In the diseased state, elevated TIMP-1 contributes to collagen preservation, tissue stiffness, and fibrosis perpetuation [46]. Consistent with this, we observed a significant upregulation of TIMP-1 in ethanol-treated cells, which was reversed by T4OL in a dose-dependent manner. This suggests that T4OL helps restore MMP/TIMP balance, which is critical for halting fibrosis progression.

In addition to fibrosis, inflammation plays a crucial role in ALD progression. IL-6 is a pro-inflammatory cytokine that is highly expressed during ethanol-induced liver injury. It contributes to neutrophil infiltration, Kupffer cell activation, and acute-phase protein synthesis via the JAK/STAT3 signaling pathway [47]. Chronic IL-6 elevation has been linked to the progression of steatohepatitis and fibrogenesis and correlates with poor outcomes in ALD patients [48,49]. Our findings revealed marked ethanol-induced IL-6 expression, which was suppressed by T4OL treatment, reflecting its anti-inflammatory potential and its ability to disrupt the IL-6/STAT3 axis.

On the oxidative stress front, GPX7 functions as part of the antioxidant defense system by reducing peroxides and maintaining redox homeostasis. Though elevated GPX7 expression may be a compensatory response to ethanol-induced ROS, persistent overexpression may also be indicative of redox imbalance and cellular stress. Recent studies have shown that GPX7 expression is upregulated in fibrotic and steatotic liver tissues and is associated with hepatic stellate cell activation [50]. In this study, GPX7 was significantly increased in ethanol-exposed HepG2 cells, indicating oxidative burden. T4OL treatment normalized GPX7 levels, supporting its antioxidant function and potential to restore redox balance. Moreover, pretreatment with different concentrations of T4OL demonstrated increased concentrations of GSH and SOD, indicating its protective effect against ethanol-mediated cellular stress and inflammation. Two key components in cellular defense against oxidative stress are SOD—a vital antioxidant enzyme in the body, and GSH—a crucial enzyme that catalyzes the breakdown of hydrogen peroxide [51]. The increased concentrations of GSH and SOD in T4OL cells reveal the antioxidant potential of the tested compound.

Studies also indicate that oxidative stress owing to excessive alcohol intake promotes HIF-1α production in hepatocytes, which induces steatosis and eventually leads to hepatic fibrosis [52]. Mice with hepatocyte-specific HIF-1α deletion have shown reduced levels of steatosis as compared to wild-type mice [52]. Our data also support previous findings and suggest that T4OL’s beneficial effects in the treatment of ALD might also involve downregulation of HIF-1α.

Collectively, these findings demonstrate that T4OL exerts a tri-modal protective effect in the context of ALD:

(1) Anti-fibrotic—by suppressing COL1A1, TGF-β1, and TIMP-1;
(2) Anti-inflammatory—by attenuating IL-6 expression;
(3) Antioxidant—by normalizing GPX7 and HIF-1α levels and increasing GSH and SOD.

These outcomes position T4OL as a multi-target therapeutic candidate capable of mitigating the complex pathology of ALD. Notably, the effectiveness of T4OL at higher concentrations (650 µM and 1.3 mM) exceeded that of SIL, a well-established hepatoprotective agent, in terms of restoring antioxidant defenses and suppressing fibrotic and inflammatory markers. This suggests that T4OL may offer superior therapeutic efficacy with a broader mechanism of action.

## 5. Conclusions

Based on the findings, it can be concluded that T4OL effectively suppresses the expression of key biomarkers associated with inflammation, oxidative stress, and fibrosis. Its hepatoprotective effects appear to stem from its ability to modulate inflammatory responses and fibrotic processes, mitigating the pathological mechanisms underlying ethanol-induced liver damage.

These combined effects demonstrate T4OL’s potential as a promising therapeutic agent for protecting the liver from ethanol-induced damage by targeting multiple pathological processes. However, further investigations are necessary to confirm its efficacy and safety beyond the cellular model.

## Figures and Tables

**Table 1:** Molecular docking analysis of T4OL and SIL.

Compound	S Score (kcal/mol)	RMSD (Å)	Atom of Compounds	Atom of Receptors	Residue of Receptor	Type of Interaction Bond	Distance (Å)	E (kcal/mol)
**NF-KB (PDB ID: 1IKN)**								
**T4OL**	−5.05	1.19	O-28	NH2	ARG 253 (A)	H-acceptor	3.26	−1.3
**SIL**	−7.98	1.14	O-54 6-ring	O CA	PRO 275 (A) THR 52 (A)	H-donor pi-H	3.08 3.93	−0.9 −0.5
**HIF-1α (PDB ID: 1H2M)**								
**T4OL**	−4.84	0.61	O-28	OE1	GLU 59 (A)	H-donor	2.89	−2.5
**SIL**	−6.52	1.86	O-47 O-49 O-54 O-56 6-ring	OD1 OE1 OE1 OE1 NE2	ASP 104 (A) GLU 105 (A) GLN 148 (A) GLN 814 (S) GLN 148 (A)	H-donor H-donor H-donor H-donor pi-H	2.82 3.13 3.09 2.84 4.27	−3.1 −1.8 −0.9 −2.9 −0.6
**TNF-α (PDB ID: 2AZ5)**								
**Terpinen-4-ol**	−4.63	1.35	O-28	O	ASN 92 (A)	H-donor	2.70	−2.1
**Silymarin**	−7.13	1.46	O-49 6-ring 6-ring	O CB CA	ARG 32 (C) ALA 35 (C) GLY 148 (C)	H-donor pi-H pi-H	2.85 3.69 3.84	−2.4 −0.5 −0.5
**COL1A1 (PDB ID: 5K31)**								
**T4OL**	−4.90	1.35	O-28	O	PRO 37 (F)	H-donor	2.82	−1.3
**SIL**	−8.42	2.02	O-49 O-54	O O	ILE 27 (F) ASP 59 (F)	H-donor H-donor	2.88 2.79	−0.5 −1.3
**MMP-1 (PDB ID: 3SHI)**								
**T4OL**	−5.40	1.85	O-28 C-24	OE2 5-ring	GLU 219 (A) HIS 218 (A)	H-donor H-pi	3.43 3.68	−0.8 −0.8
**SIL**	−7.86	1.72	O-47 O-49 O-49	OE1 OE1 N	GLU 209 (A) GLU 201 (A) ARG 202 (A)	H-donor H-donor H-acceptor	2.89 2.87 3.35	−4.5 −4.1 −0.9
**TIMP-1 (PDB ID: 1UEA)**								
**T4OL**	−4.86	1.73	O-28 O-28	O N	VAL 102 (D) VAL 102 (D)	H-donor H-acceptor	2.84 3.05	−1.6 −2.2
**SIL**	−8.33	1.33	O-54 O-56 6-ring 6-ring 6-ring 6-ring	O O N CB CD2 N	GLU 216 (A) TYR 220 (A) HIS 224 (A) LEU 226 (A) LEU 226 (A) THR 227 (A)	H-donor H-donor pi-H pi-H pi-H pi-H	2.73 3.02 4.30 4.36 4.15 4.43	−2.3 −3.6 −0.8 −0.7 −0.5 −0.5

## Data Availability

The datasets used and/or analyzed during this study are available from the corresponding author upon reasonable request.

## Supplementary Materials

The following supporting information can be downloaded at: https://www.mdpi.com/article/10.3390/medicina61061048/s1, Figure S1: 2D structure of Ligand (T4OL); Figure S2: Representation of 3D crystal structure of proteins by Discovery studio. (A) Human IKB-α/NF-κB complex (B) Human HIF-1α (C) Human TNF-α (D) Human COL1A1 (E) Human MMP-1 (F) Human MMP-3/TIMP-1 complex; Figure S3: T4OL did not affect cell viability at doses ranging from 13–1300 µM. HepG2 cells were treated with different concentrations of T4OL (13, 130, 650, 1300, 2600 µM) for 24 h. T4OL at concentration 2600 µM significantly attenuated the viability of HepG2 cells. One-way ANOVA and postHoc test, n = 4, *** ≤ 0.001; Figure S4: CV assay demonstrating hepatoprotective potential of T4OL. HepG2 cells were pretreated with T4OL for 24 h, followed by 24 h intoxication with 8% ethanol. Cells were further treated with 0.2% crystal violet solution and incubated for 15 minutes. After incubation, the dye was dissolved by adding 100 µL of 1% SDS and the absorbance was measured at 590nm. Crystal violet assay results revealed significant hepatoprotective effect of T4OL against ethanol induced injury. One way ANOVA followed by Tukey’s multiple comparison test; (n = 4), *** ≤ 0.001, ** ≤ 0.01, * ≤ 0.05; Table S1: Binding sites prediction of proteins.

## References

[1] Navarro V.J., Senior J.R. (2006). Drug-related hepatotoxicity. N. Engl. J. Med..

[2] Sharma S.K., Balamurugan A., Saha P.K., Pandey R.M., Mehra N.K. (2002). Evaluation of clinical and immunogenetic risk factors for the development of hepatotoxicity during antituberculosis treatment. Am. J. Respir. Crit. Care Med..

[3] Seitz H.K., Bataller R., Cortez-Pinto H., Gao B., Gual A., Lackner C., Mathurin P., Mueller S., Szabo G., Tsukamoto H. (2018). Alcoholic liver disease. Nat. Rev. Dis. Primer.

[4] Seitz H.K., Neuman M.G. (2021). The history of alcoholic liver disease: From an unrecognized disease to one of the most frequent diseases in hepatology. J. Clin. Med..

[5] Kong L.Z., Chandimali N., Han Y.H., Lee D.H., Kim J.S., Kim S.U., Kim T.D., Jeong D.K., Sun H.N., Lee D.S. (2019). Pathogenesis, Early Diagnosis, and Therapeutic Management of Alcoholic Liver Disease. Int. J. Mol. Sci..

[6] Singal A.K., Bataller R., Ahn J., Kamath P.S., Shah V.H. (2018). ACG Clinical Guideline: Alcoholic Liver Disease. Am. J. Gastroenterol..

[7] Gao B., Bataller R. (2011). Alcoholic liver disease: Pathogenesis and new therapeutic targets. Gastroenterology.

[8] Morley K.C., Leung S., Baillie A., Haber P.S. (2013). The efficacy and biobehavioural basis of baclofen in the treatment of alcoholic liver disease (BacALD): Study protocol for a randomised controlled trial. Contemp. Clin. Trials.

[9] Chen Y., Singh S., Matsumoto A., Manna S.K., Abdelmegeed M.A., Golla S., Murphy R.C., Dong H., Song B.J., Gonzalez F.J. (2016). Chronic Glutathione Depletion Confers Protection against Alcohol-induced Steatosis: Implication for Redox Activation of AMP-activated Protein Kinase Pathway. Sci. Rep..

[10] Zhao H., Zhao C., Dong Y., Zhang M., Wang Y., Li F., Li X., McClain C., Yang S., Feng W. (2015). Inhibition of miR122a by Lactobacillus rhamnosus GG culture supernatant increases intestinal occludin expression and protects mice from alcoholic liver disease. Toxicol. Lett..

[11] Muriel P., Rivera-Espinoza Y. (2008). Beneficial drugs for liver diseases. J. Appl. Toxicol..

[12] Osna N.A., Donohue T.M., Kharbanda K.K. (2017). Alcoholic Liver Disease: Pathogenesis and Current Management. Alcohol. Res..

[13] Nault J.C., Bioulac–Sage P., Zucman–Rossi J. (2013). Reviews in basic and clinical gastroenterology and hepatology. Gastroenterology.

[14] Abe R., Ohtani K. (2013). An ethnobotanical study of medicinal plants and traditional therapies on Batan Island, the Philippines. J. Ethnopharmacol..

[15] Atanasov A.G., Waltenberger B., Pferschy-Wenzig E.M., Linder T., Wawrosch C., Uhrin P., Temml V., Wang L., Schwaiger S., Heiss E.H. (2015). Discovery and resupply of pharmacologically active plant-derived natural products: A review. Biotechnol. Adv..

[16] Yadav E., Rao R. (2016). A promising bioactive component terpinen-4-ol: A review. Int. J. Pharmacog..

[17] Bakkali F., Averbeck S., Averbeck D., Idaomar M. (2008). Biological effects of essential oils—A review. Food Chem. Toxicol..

[18] Brand C., Ferrante A., Prager R.H., Riley T.V., Carson C.F., Finlay-Jones J.J., Hart P.H. (2001). The water-soluble components of the essential oil of *Melaleuca alternifolia* (tea tree oil) suppress the production of superoxide by human monocytes, but not neutrophils, activated in vitro. Inflamm. Res..

[19] Calcabrini A., Stringaro A., Toccacieli L., Meschini S., Marra M., Colone M., Salvatore G., Mondello F., Arancia G., Molinari A. (2004). Terpinen-4-ol, the main component of *Melaleuca alternifolia* (tea tree) oil inhibits the in vitro growth of human melanoma cells. J. Invest. Dermatol..

[20] Gfeller D., Grosdidier A., Wirth M., Daina A., Michielin O., Zoete V. (2014). SwissTargetPrediction: A web server for target prediction of bioactive small molecules. Nucleic Acids Res..

[21] Lagunin A., Ivanov S., Rudik A., Filimonov D., Poroikov V. (2013). DIGEP-Pred: Web service for in silico prediction of drug-induced gene expression profiles based on structural formula. Bioinformatics.

[22] Pogodin P.V., Lagunin A.A., Filimonov D.A., Poroikov V.V. (2015). PASS Targets: Ligand-based multi-target computational system based on a public data and naive Bayes approach. SAR QSAR Env. Res..

[23] Gallo K., Goede A., Preissner R., Gohlke B.O. (2022). SuperPred 3.0: Drug classification and target prediction-a machine learning approach. Nucleic Acids Res..

[24] Wang X., Shen Y., Wang S., Li S., Zhang W., Liu X., Lai L., Pei J., Li H. (2017). PharmMapper 2017 update: A web server for potential drug target identification with a comprehensive target pharmacophore database. Nucleic Acids Res..

[25] Ochoa D., Hercules A., Carmona M., Suveges D., Gonzalez-Uriarte A., Malangone C., Miranda A., Fumis L., Carvalho-Silva D., Spitzer M. (2021). Open Targets Platform: Supporting systematic drug-target identification and prioritisation. Nucleic Acids Res..

[26] Jia A., Xu L., Wang Y. (2021). Venn diagrams in bioinformatics. Brief. Bioinform..

[27] Tang D., Chen M., Huang X., Zhang G., Zeng L., Zhang G., Wu S., Wang Y. (2023). SRplot: A free online platform for data visualization and graphing. PLoS ONE.

[28] Shannon P., Markiel A., Ozier O., Baliga N.S., Wang J.T., Ramage D., Amin N., Schwikowski B., Ideker T. (2003). Cytoscape: A software environment for integrated models of biomolecular interaction networks. Genome Res..

[29] Mering C.v., Huynen M., Jaeggi D., Schmidt S., Bork P., Snel B. (2003). STRING: A database of predicted functional associations between proteins. Nucleic Acids Res..

[30] Consortium G.O. (2006). The gene ontology (GO) project in 2006. Nucleic Acids Res..

[31] Gao Y.-D., Huang J.-F. (2011). An extension strategy of Discovery Studio 2.0 for non-bonded interaction energy automatic calculation at the residue level. Dongwuxue Yanjiu.

[32] Huxford T., Huang D.B., Malek S., Ghosh G. (1998). The crystal structure of the IkappaBalpha/NF-kappaB complex reveals mechanisms of NF-kappaB inactivation. Cell.

[33] Sharma U., Carrique L., Vadon-Le Goff S., Mariano N., Georges R.N., Delolme F., Koivunen P., Myllyharju J., Moali C., Aghajari N. (2017). Structural basis of homo- and heterotrimerization of collagen I. Nat. Commun..

[34] Tian W., Chen C., Lei X., Zhao J., Liang J. (2018). CASTp 3.0: Computed atlas of surface topography of proteins. Nucleic Acids Res..

[35] Maqbool T., Awan S.J., Malik S., Hadi F., Shehzadi S., Tariq K. (2019). In-Vitro Anti-Proliferative, Apoptotic and Antioxidative Activities of Medicinal Herb Kalonji (*Nigella sativa*). Curr. Pharm. Biotechnol..

[36] Lee J.Y., Kim H., Jeong Y., Kang C.H. (2021). Lactic Acid Bacteria Exert a Hepatoprotective Effect against Ethanol-Induced Liver Injury in HepG2 Cells. Microorganisms.

[37] Pouresmaeil V., Al Abudi A.H., Mahimid A.H., Sarafraz Yazdi M., Es-Haghi A. (2023). Evaluation of Serum Selenium and Copper Levels with Inflammatory Cytokines and Indices of Oxidative Stress in Type 2 Diabetes. Biol. Trace Elem. Res..

[38] Valko M., Leibfritz D., Moncol J., Cronin M.T., Mazur M., Telser J. (2007). Free radicals and antioxidants in normal physiological functions and human disease. Int. J. Biochem. Cell Biol..

[39] Kumar C.H., Ramesh A., Kumar J.S., Ishaq B.M. (2011). A review on hepatoprotective activity of medicinal plants. Int. J. Pharm. Sci. Res..

[40] Gan Y., Tong J., Zhou X., Long X., Pan Y., Liu W., Zhao X. (2021). Hepatoprotective Effect of Lactobacillus plantarum HFY09 on Ethanol-Induced Liver Injury in Mice. Front. Nutr..

[41] Zhang S., Gong Y., Xiao J., Chai Y., Lei J., Huang H., Xiang T., Shen W. (2018). A COL1A1 promoter-controlled expression of TGF-β soluble receptor inhibits hepatic fibrosis without triggering autoimmune responses. Dig. Dis. Sci..

[42] Hayashi M., Nomoto S., Hishida M., Inokawa Y., Kanda M., Okamura Y., Nishikawa Y., Tanaka C., Kobayashi D., Yamada S. (2014). Identification of the collagen type 1 alpha 1 gene (COL1A1) as a candidate survival-related factor associated with hepatocellular carcinoma. BMC Cancer.

[43] Gressner A., Weiskirchen R. (2006). Modern pathogenetic concepts of liver fibrosis suggest stellate cells and TGF-β as major players and therapeutic targets. J. Cell Mol. Med..

[44] Dooley S., ten Dijke P. (2012). TGF-beta in progression of liver disease. Cell Tissue Res..

[45] Hemmann S., Graf J., Roderfeld M., Roeb E. (2007). Expression of MMPs and TIMPs in liver fibrosis—A systematic review with special emphasis on anti-fibrotic strategies. J. Hepatol..

[46] Lin J., Deng C., Peng Y., Zheng J., Wei L., Shi Y., Gong Z., Hu G. (2019). Dynamic Changes in MMP1 and TIMP1 in the Antifibrotic Process of Dahuang Zhechong Pill in Rats with Liver Fibrosis. Open Chem..

[47] Hou X., Yin S., Ren R., Liu S., Yong L., Liu Y., Li Y., Zheng M.H., Kunos G., Gao B. (2021). Myeloid-Cell-Specific IL-6 Signaling Promotes MicroRNA-223-Enriched Exosome Production to Attenuate NAFLD-Associated Fibrosis. Hepatology.

[48] Scarlata G.G.M., Colaci C., Scarcella M., Dallio M., Federico A., Boccuto L., Abenavoli L. (2024). The Role of Cytokines in the Pathogenesis and Treatment of Alcoholic Liver Disease. Diseases.

[49] Schmidt-Arras D., Rose-John S. (2016). IL-6 pathway in the liver: From physiopathology to therapy. J. Hepatol..

[50] Yao J., Chen X., Liu Z., Zhang R., Zhang C., Yang Q., Yao P., Jiang Q., Wu J., Zhao S. (2021). The increasing expression of GPX7 related to the malignant clinical features leading to poor prognosis of glioma patients. Chin. Neurosurg. J..

[51] Xiao Z., Yu X., Zhang S., Liang A. (2022). The Expression Levels and Significance of GSH, MDA, SOD, and 8-OHdG in Osteochondral Defects of Rabbit Knee Joints. Biomed. Res. Int..

[52] Bostrom P., Magnusson B., Svensson P.A., Wiklund O., Boren J., Carlsson L.M., Stahlman M., Olofsson S.O., Hulten L.M. (2006). Hypoxia converts human macrophages into triglyceride-loaded foam cells. Arter. Thromb. Vasc. Biol..

