# Supplementary material for: Terpinen-4-ol Targets HIF-1α/TGF-β1/TNF-α Axis to Attenuate Ethanol-Induced Hepatotoxicity: Network Pharmacology and In Vitro Validation
Source: Medicina (Kaunas). 2025 Jun 6;61(6):1048. doi: 10.3390/medicina61061048 (PMC12195375; doi:10.3390/medicina61061048)
Supplement: Supplementary file 1 [file medicina-61-01048-s001.zip › medicina-3615276-supplementary.pdf]

Supplementary material

Table S1: Binding sites prediction of proteins

**Proteins (PDB ID)**	**Surface area (Å^2)**	**Volume (Å^3)**
Human IKB-α/NFKB complex (1IKN)	1133.582	943.278
Human HIF-1α (1H2M)	654.287	1214.922
Human TNF-α (2AZ5)	2743.256	4514.068
Human COL1A1 (5K31)	7249.891	1299.264
Human MMP-1 (3SHI)	201.599	126.171
Human MMP-3/TIMP-1 complex (1UEA)	417.283	592.610

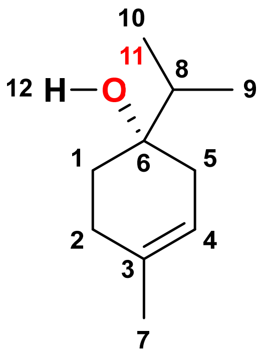

Figure S1: 2D structure of Ligand (T4OL)

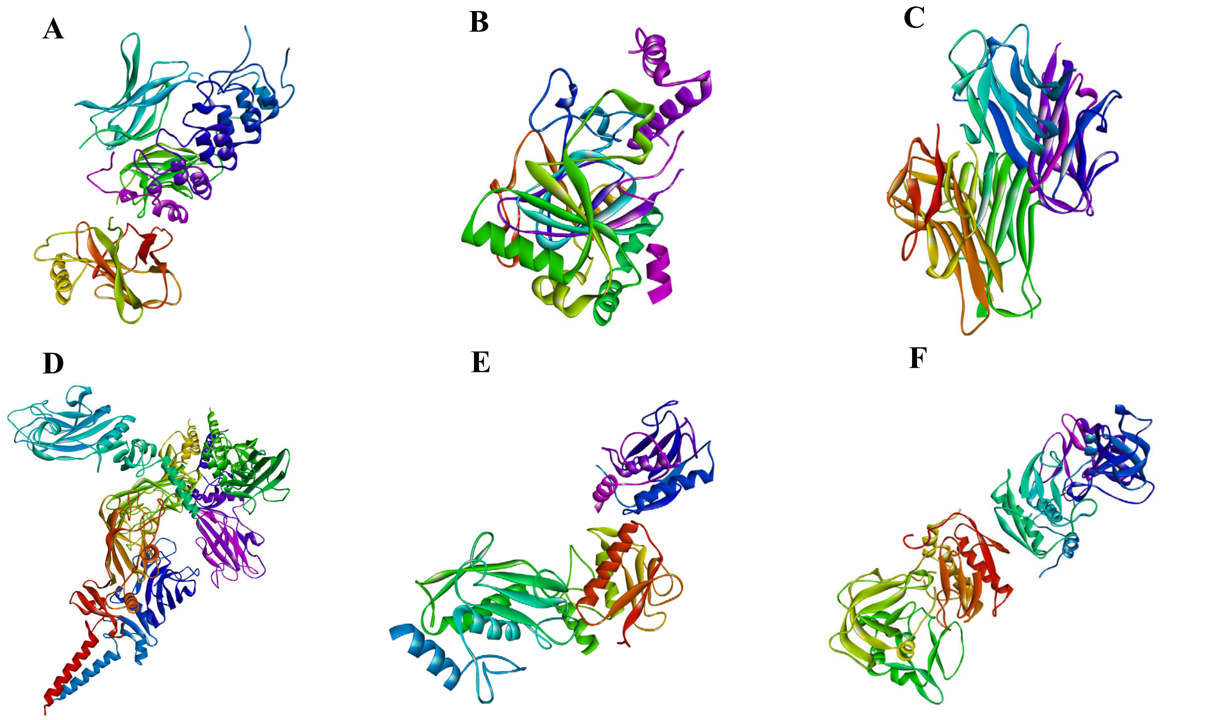

Figure S2: Representation of 3D crystal structure of proteins by Discovery studio. A) Human IKB-α/NF-κB complex B) Human HIF-1α C) Human TNF-α D) Human COL1A1 E) Human MMP-1 F) Human MMP-3/TIMP-1 complex

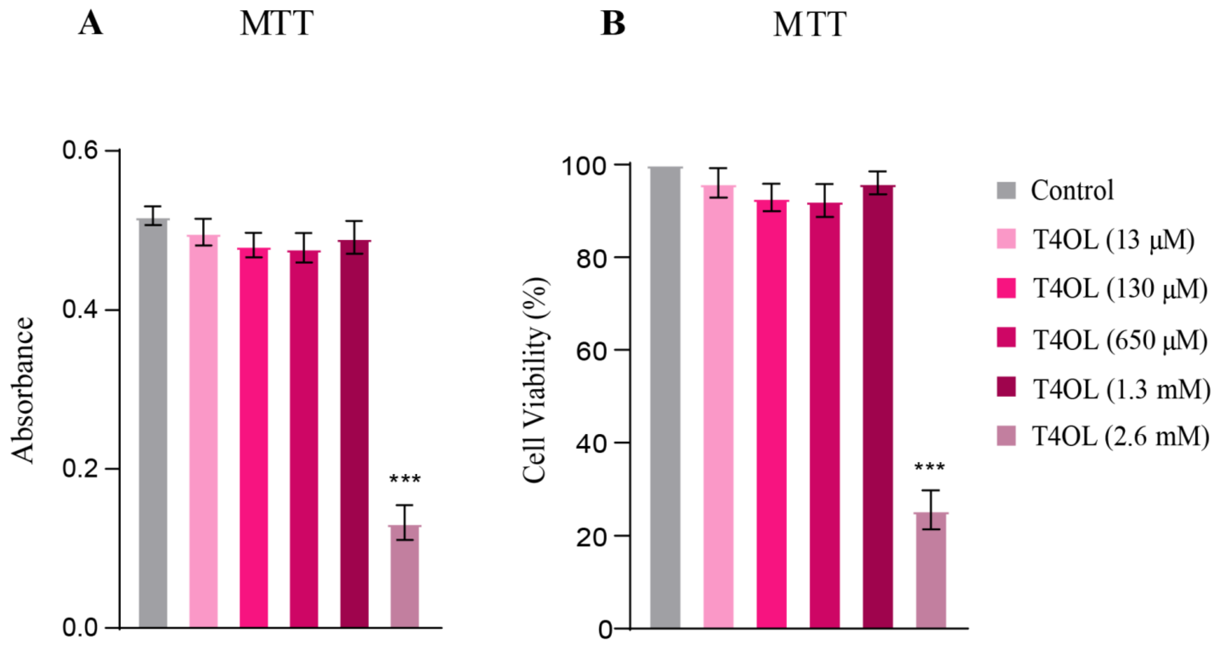

Figure S3: T4OL did not affect cell viability at doses ranging from 13 – 1300 µM. HepG2 cells were treated with different concentrations of T4OL (13, 130, 650, 1300, 2600 µM) for 24 h. T4OL at concentration 2600 µM significantly attenuated the viability of HepG2 cells. One-way ANOVA and postHoc test, $n = 4$, ***≤ 0.001

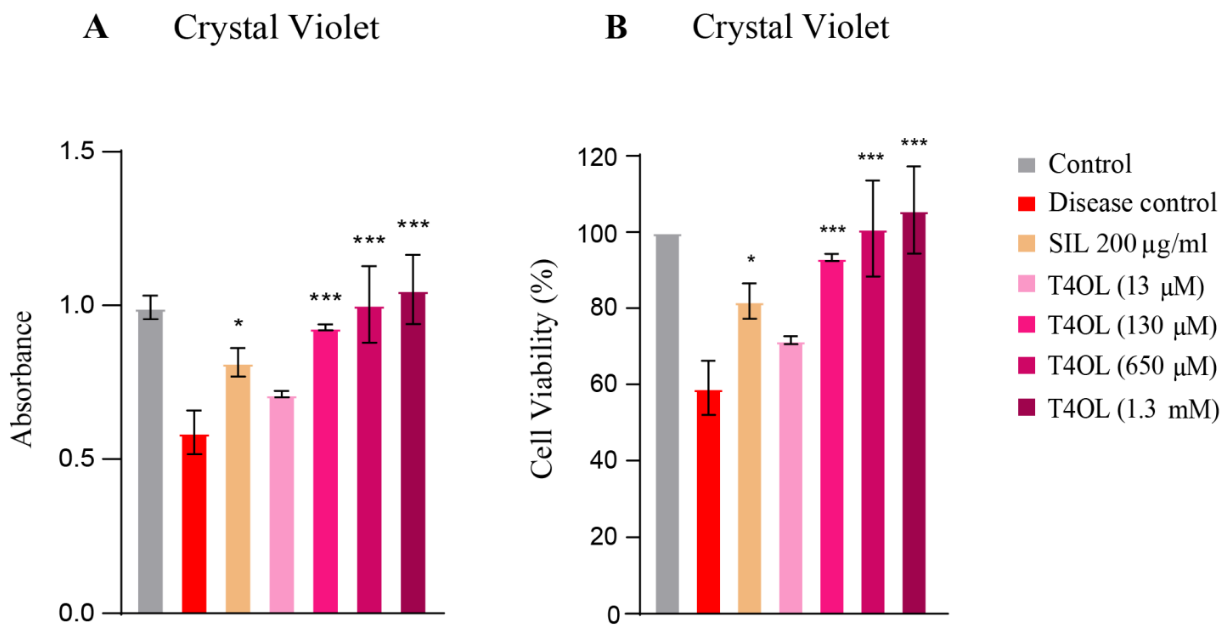

Figure S4: CV assay demonstrating hepatoprotective potential of T4OL. HepG2 cells were pretreated with T4OL for 24 h, followed by 24 h intoxication with 8% ethanol. Cells were further treated with 0.2% crystal violet solution and incubated for 15 minutes. After incubation, the dye was dissolved by adding 100 µl of 1% SDS and the absorbance was measured at 590nm. Crystal violet assay results revealed significant hepatoprotective effect of T4OL against ethanol induced injury. One way ANOVA followed by Tukey's multiple comparison test; (n = 4), *** ≤ 0.001, ** ≤ 0.01, * ≤ 0.05.
